# Association of Migraine and Ischemic Heart Disease: A Review

**DOI:** 10.7759/cureus.5719

**Published:** 2019-09-21

**Authors:** Aisha Saeed, Kiran F Rana, Zain I Warriach, Muhammad Ali Tariq, Bilal Haider Malik

**Affiliations:** 1 Family Medicine, California Institute of Behavioral Neurosciences and Psychology, Fairfield, USA; 2 Psychiatry, California Institute of Behavioral Neurosciences and Psychology, Fairfield, USA; 3 Internal Medicine, California Institute of Behavioral Neurosciences and Psychology, Fairfield, USA

**Keywords:** migraine, headache, cardiovascular diseases, ischemic heart disease, myocardial ischemia, migraine disorders, coronary artery disease, migraine and ischemic heart disease

## Abstract

Many new studies have shown an association between migraine and ischemic heart disease, and the association seems to be multi-factorial. This article reviews what is already known about this linkage and further investigates if migraine is a risk factor for cardiovascular disease.

The literature search for this article was performed primarily using PubMed as the search engine. Only those articles that assessed migraine as exposure and cardiovascular events as outcomes were included. Also, articles only from the last five years with full-text and human studies were reviewed.

Based on our investigation and as indicated by previous studies, migraine headache is associated with different kinds of cardiovascular events. Healthcare providers need to be aware of this association so that they can assess and manage their migraine patients better.

## Introduction and background

The healthcare cost associated with migraine is about $36 billion annually in the United States, which is expected to rise in the future. While the total expenditure on cardiovascular disease was $555 billion in the US in the year 2016, and this figure is projected to go up to $1.1 trillion by 2035.

Migraine is a neurological disease affecting approximately one billion people worldwide and is considered as the 6th most disabling disease in the world [1-3]. It is more common in women than men and affects over 30% of women between the ages of 25 and 55 [4,5]. Due to the throbbing nature of the disease, it is common to think of migraines to have a vascular etiology, when in fact there are three different theories including vascular theory, neurovascular theory and cortical spreading depolarization (CSD) [6,7]. Migraine is an inherent disease having genetic association of about 42% [8]. Certain environmental factors can bring about or worsen a migraine attack including diet, stress, alcohol, smoking, sleep disturbance, hormonal changes, sensory stimuli (light smells or noises), weather change and certain foods [9,10].

Numerous studies have shown that migraineurs have a higher chance of experiencing an ischemic stroke, a risk two times more common in patients experiencing migraine with aura. Aura is a reversible set of neurologic symptoms that usually comes before a migraine attack, is mostly visual and lasts about 5-60 minutes. Aura is only seen in 1/4th of the population suffering from migraine [11,12].

Migraine (aura) can be considered one of the risk factors for ischemic heart disease (IHD) according to new emerging evidence [6,13]. Although the exact relationship between migraine and ischemic heart disease is still not completely known, some studies suggest the association to be multi-factorial, suggesting different mechanisms for different cardiovascular events [14]. One of the proposed mechanisms seems to be endothelial dysfunction [15-17].

A higher prevalence of patent foramen ovale in patients suffering from migraine (aura), increased release of inflammatory markers, and the use of non-steroid anti-inflammatory drugs (NSAID) in these patients can further increase the likelihood of myocardial infarction, venous thromboembolism and atrial fibrillation [18-22]. Some studies also suggest the association between migraine and cardiovascular events to be genetic in origin, as shown by a genome-wide analysis study [23-25].

There is an urgent need to understand the association between migraine and ischemic heart disease (IHD) so that improved adjustments can be made in the treatment of patients suffering from migraine. In this review, we are going to investigate further the population most at risk of cardiovascular events with migraine headache. This article will help guide physicians to manage patients better and decide what preventive strategies need to be used to minimize the risk of future cardiovascular events in migraineurs.

## Review

Methods

The electronic search was performed mostly using PubMed as the search engine. Some articles were also taken from Google Scholar, Embase, MEDLINE, and grey literature. The search was performed using the Mesh terms and keywords “Migraine”, “Ischemic Heart Disease (IHD)”, “Cardiovascular disease (CVD)”, “Migraine and IHD”, “Headache and Cardiovascular disease”, “Myocardial ischemia”, “Migraine disorder” and “Coronary artery disease”. The number of articles yielded with each keyword and mesh term is shown in Tables 1, 2. No guidelines or statements were taken from preferred reporting items for systematic reviews and meta-analyses (PRISMA) or meta-analyses of observational studies in epidemiology (MOOSE).

**Table 1:** Search results with simple keywords

KEYWORDS	DATABASE	NUMBER OF RESULTS
Cardiovascular Disease	PubMed	2417123
Ischemic Heart Disease	PubMed	486120
Headache	PubMed	91338
Migraine	PubMed	37409
Headache and cardiovascular disease	PubMed	16616
Migraine and Ischemic Heart Disease	PubMed	592

**Table 2:** Search results with mesh keywords

MeSH KEYWORDS	DATABASE	NUMBER OF RESULTS
Myocardial Ischemia	PubMed	470064
Migraine Disorder	Google Scholar	291000
Coronary Artery Disease	PubMed	160695

All types of studies and review articles evaluating cardiovascular outcomes in migraine patients were included. Other selection criteria used in this review were: full-text articles, articles published in the last five years, and articles with humans as subjects. Some facts were also taken from grey literature.

Everything was done scientifically and ethically. We did not use any quality assessment tool or perform any statistical analyses.

Results

The initial search was performed with PubMed using the following keywords: ‘Cardiovascular Disease (CVD)' which generated 2417123 articles, ‘Ischemic Heart Disease' brought up 486120 studies, ‘Headache' resulted in 91338 while search with the word ‘Migraine' resulted in 37409 studies. ‘Headache and Cardiovascular Disease' generated 16616 articles, and a search with keyword ‘Migraine and Ischemic Heart Disease' generated 592 results.

Of these 592 articles, 513 were excluded based on the inclusion/exclusion criteria (full-text, five-years, and human). Among the 79 studies that were reviewed, 40 were excluded from the revision of titles and abstract, four did not evaluate the outcome of interest, and one article was not available. Thirty-four studies were included in the final analysis. There are about eight full articles. The included studies are from five countries. The search was performed without any language restriction.

One of the included articles is a cohort study done in the Danish population, which studied 51032 migraine patients and 510320 people from the general population matched on age, sex, and calendar year. These patients were followed up for 19 years, and the study showed that migraine is positively linked with myocardial infarction [14].

A meta-analysis of 16 cohort studies included 1,152,407 subjects with a follow-up duration of up to 26 years [26]. The included studies were adjusted for age and co-morbidities like hypertension, diabetes, and hyperlipidemia. The subjects were assessed either through questionnaires or hospital records, and the final result showed that migraine with aura is a risk factor for major cardiovascular and cerebrovascular events [26].

Two review articles show a strong association between migraine and cardiovascular events [7,27]. Three studies illustrate the association is higher in women suffering from migraine with aura [16,27,28]. A systemic review and meta-analysis of observational studies indicates that migraine is related to myocardial infarction (MI) and angina [15]. 

Few studies show inconsistent results; one found heterogeneity among reviewed studies and suggested the idea that migraine with aura increases cardiovascular events [29,30].

We agree with the majority of these articles that there is an association between migraine and IHD (Table 3).

**Table 3:** Researches correlating migraine and CVD MI- Myocardial infarction, CVD- Cardiovascular disease, PFO- Patent foramen ovale, ASD- Arterial septal defect, MVP- Mitral valve prolapse, MA- Migraine with aura

AUTHOR	YEAR	COUNTRY	OUTCOME
Sacco et al. [15]	2015	Italy	Migraine is associated with MI and angina and can be considered as a risk factor for CVD.
Alqaqa [7]	2016	USA	There is an increased risk of stroke, angina, arrhythmia, PFO, ASD, and MVP with MA. The physicians should be aware of modifiable cardiovascular risk factors.
Linstra et al. [27]	2017	The Netherlands	Migraine can be appropriately considered as an overall risk factor for CVD, especially in young women with aura.
Rambarat et al. [16]	2017	USA	Those women who have a history of migraine headache and are showing the signs and symptoms of ischemia are at risk of CVD.
Adelborg et al. [14]	2018	Denmark	Migraine ought to be considered a constant risk factor for most cardiovascular diseases in both men and women.
Mahmoud et al. [26]	2018	USA	There is an increased risk of cardiovascular and cerebrovascular events with MA.

**Figure 1:**
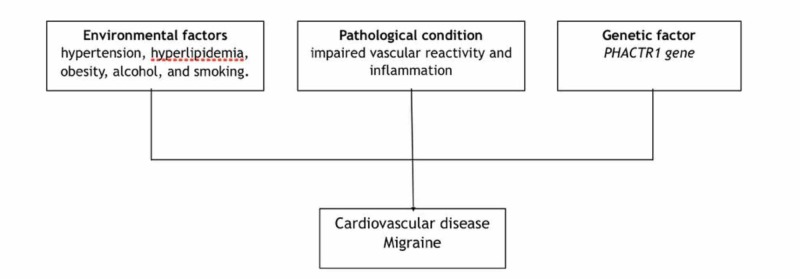
Shared risk factors for migraine and CVD CVD- Cardiovascular disease PHACTR 1-  Phosphatase and actin regulator 1

Some risk factors are commonly shared between migraine and CVD, as indicated in Figure 1 and explained in detail later in our article.

Discussion

The link between migraine and stroke has already been established in literature so we will only highlight the association of migraine and IHD. Our review indicates that migraine is associated with several cardiovascular diseases and this connection is found to be stronger in patients who experience aura than in those without aura. Below are some of the mechanisms involved in the link between migraine and IHD.

Physiological/Pathophysiological Process

To understand the relationship between migraine and cardiovascular events, it is important to understand the mechanisms involved in migraine. Three major theories for the pathophysiology of migraine have been proposed in the literature, including the vascular theory, the neurovascular theory, and the theory of cortical spreading depression (CSD) [7]. The ischemic changes that occur during a migraine attack seem to spread to other organs as well, especially the heart.

The vascular theory suggests that vasoconstriction is responsible for the aura while vasodilatation is responsible for headache during a migraine attack. The neurovascular theory is to be blamed for the neurogenic and vascular alteration in cerebral perfusion that produces migraine episode. Cortical spreading depression can further predispose the brain to cerebral hypoperfusion and arterial ischemia.

Is the proposed pathophysiological mechanism true, or are there other mechanisms causing this association between migraine and IHD?

Biochemical Process

Oxidative stress has also been considered as an important factor in the pathogenesis of migraine headaches and can cause alteration in the cerebral blood flow [31]. A marker for oxidative stress called 4-hydroxy-2-nonenal (HNE) has also been found to be elevated in migraineurs and has been suggested to be involved in the pathogenesis of IHD [32]. More importantly, oxidative stress has been associated with endothelial dysfunction, atherosclerosis, and increased vascular risk.

Circulating endothelial progenitor cells (EPC) are responsible for neo-angiogenesis after ischemic insult, and their number and function is found to decline in migraine patients [17,31]. Deficiency or loss of EPC's can interrupt the balance between endothelial damage and restorative function, leading to endothelial dysfunction, atherosclerosis, and elevated vascular risk. In addition, increased levels of prothrombin, von Willebrand factor, and endothelin also increase cardiovascular risk.

Environmental Factors

Many studies have clearly shown that a change in environmental factors often triggers migraine attacks. A large systemic review has established that the most common triggers for migraine attacks are stress, auditory stimuli, fatigue, fasting, weather changes, and menses [9].

Some studies suggest that increased risk of cardiovascular events in migraine patients can be ascribed to a higher occurrence of cardiovascular risk factors, such as smoking, alcohol, high salt intake (hypertension) and obesity (hypercholesterolemia) [26]. Smoking, especially in women migraine patients, increases the risk of stroke [33]. Advice on healthy lifestyle, mainly smoking cessation, control of hypertension (HTN), and maintenance of normal body weight, may aid in controlling some of the modifiable risk factors. 

If we control these modifiable cardiovascular risk factors in migraine patients, will they still experience the cardiovascular events?

Genetic Factors

If migraine and coronary artery disease (CAD) share the same pathological process, they likely share genetic risk as well. Winsvold et al. determined the genetic overlap between migraine and CAD by performing analyses based on three large genome-wide association study (GWAS) meta-analyses of migraine and coronary artery disease [23]. One shared risk locus was identified as encoding phosphatase and actin regulator 1 (PHACTR1) gene. This is a genome-wide significant risk locus for both migraine and CAD. This protein phosphatase 1 binding protein is highly expressed in the brain where it regulates synaptic activity and dendritic morphology. It is also expressed in arteries and plays an important role in the regulation of endothelial function and is associated with altered vasomotor tone [34].

More work needs to be done in order to improve our understanding of the genetic association between migraine and CAD. More importantly, how this genetic association plays a role in the pathogenic and vascular mechanism of migraine? Is there more than one gene involved in this association?

Hormones

Sex hormones seem to be related to migraine, and this may be responsible for the gender difference seen in migraine patients. Fluctuating levels of sex hormones, especially estrogen, predisposes females to migraine attacks by increasing cortical excitability. Estrogen has a major influence on vascular health as it is involved in both thrombotic as well as vasodilatory mechanisms. However, the role of estrogen in causing IHD in migraine patients remains unclear because of the complex mechanisms involved [27,35].

Recently, the European Headache Federation (EHF) and the European Society of Contraception and Reproductive Health (ESC) has published a statement regarding the use of hormonal contraceptives in female migraineurs [36]. In particular, the combination of smoking and oral contraceptives should be avoided in all cases in young women with migraine (aura) as this increases the risk for ischemic stroke [33].

Although the American Heart Association (AHA) has also suggested practicing caution when prescribing oral contraceptive pills (OCP), there is inadequate evidence for this recommendation [37].

Currently, there is not enough evidence to support changes in medication that may decrease the cardiovascular risk of migraine in women. Further studies should find the exact mechanism behind the gender difference in CVD risk in migraine patients.

Miscellaneous

The mechanism by which migraine increases CVD risk is multi-factorial. Different mechanisms are involved between migraine and different cardiovascular outcomes. A number of studies have shown that the patency of foramen ovale (PFO) is higher in migraine with aura which can lead to paradoxical emboli reaching the cerebral or coronary vasculature causing a stroke or myocardial infarction respectively [18]. Some clinical trials have shown that the closure of PFO can reduce the risk of stroke, but the PFO closure for migraine headache remains controversial.

Migraine patients often use non-steroid anti-inflammatory drugs (NSAIDs), which is suspected to be associated with a higher risk of myocardial infarction, venous thromboembolism (VTE) and atrial fibrillation [19,21,22]. Although currents guidelines do not recommend the use of aspirin and clopidogrel in the prophylaxis of migraine, their efficacy in preventing migraine is still under study [38].

Physicians should be aware of this association and should prescribe NSAIDs with caution, especially in patients who have a higher risk of VTE. In addition, immobilization due to migraine attacks can further increase the likelihood of VTE.

Should the physicians stop prescribing NSAIDS to migraine patients because of their cardiovascular risk profile?

What is new on the horizon?

· What mechanisms other than those proposed by previous studies are inflicting the association between migraine headache and IHD?

· If we manage the cardiovascular risk factor in migraine patients, are they still likely to experience cardiovascular events?

· The literature supports the proposal that migraine patients experience cardiovascular changes. Can it be the other way round too? Can migraine also occur in a patient with a history of IHD?

· Careful lifestyle advice like maintaining ideal body weight and smoking cessation should be given to patients who are at risk of developing CVD.

· More work must be done, to enhance our understanding of the genetic association between migraine headache and CVD.

· Researchers need to find out if any changes in medication can be beneficial for migraine patients.

Limitations

Although most of the articles used in our review were of high quality, there were some limitations that we faced. Firstly, we did not have full access to all the articles as some of the literature was paid. Secondly, not all articles were in the English language, so we faced a language barrier during our research. Thirdly, we could not access all the research engines as they were available only to members. Fourth, some of the studies have shown heterogeneity in results that should not be ignored. Lastly, we did not consider the association between migraine and other vascular disorders, which has been mentioned in some of the studies.

## Conclusions

Based on our research, we found out that migraine headache is linked with IHD. Migraine with aura is, in fact, strongly associated with adverse cardiovascular outcomes such as myocardial infarction, angina, and arrhythmia. Migraine, therefore, should be considered as an overall risk factor for cardiovascular disease. Physicians should educate the patients regarding this association and advise lifestyle modification to reduce this risk.
